# AG1^®^, a Novel Synbiotic, Maintains Gut Barrier Function following Inflammatory Challenge in a Caco-2/THP1-Blue™ Co-Culture Model

**DOI:** 10.3390/microorganisms12071263

**Published:** 2024-06-21

**Authors:** Philip A. Sapp, Jeremy R. Townsend, Trevor O. Kirby, Marlies Govaert, Cindy Duysburgh, Lynn Verstrepen, Massimo Marzorati, Tess M. Marshall, Ralph Esposito

**Affiliations:** 1 Research, Nutrition, and Innovation, AG1, Carson City, NV 89701, USA; 2 Health & Human Performance, Concordia University Chicago, River Forest, IL 60305, USA; 3 ProDigest BVBA, B-9052 Ghent, Belgium; 4 Center of Microbial Ecology and Technology (CMET), Ghent University, B-9000 Ghent, Belgium; 5 Department of Nutrition, Food Studies, and Public Health, New York University-Steinhardt, New York, NY 10003, USA

**Keywords:** gut barrier, gut integrity, dietary supplement, synbiotic, foundational nutrition

## Abstract

Nutritional interventions to reduce gastrointestinal (GI) permeability are of significant interest to physically active adults and those experiencing chronic health conditions. This in vitro study was designed to assess the impact of AG1, a novel synbiotic, on GI permeability following an inflammatory challenge. Interventions [AG1 (vitamins/minerals, pre-/probiotics, and phytonutrients) and control (control medium)] were fed separately into a human GI tract model (stomach, small intestine, and colon). In the colonic phase, the GI contents were combined with fecal inocula from three healthy human donors. GI permeability was evaluated with transepithelial electrical resistance (TEER) in a Caco-2 (apical)/THP1-Blue™ (basolateral) co-culture model. The apical side received sodium butyrate (positive control) or Caco-2 complete medium (negative control) during baseline testing. In the 24 h experiment, the apical side received colonic simulation isolates from the GI model, and the basolateral side was treated with Caco-2 complete medium, then 6 h treatment with lipopolysaccharide. TEER was assessed at 0 h and 24 h, and inflammatory markers were measured at 30 h in triplicate. Paired samples *t*-tests were used to evaluate endpoint mean difference (MD) for AG1 vs. control. TEER was higher for AG1 (mean ± SD: 99.89 ± 1.32%) vs. control (mean ± SD: 92.87 ± 1.22%) following activated THP1-induced damage [MD: 7.0% (*p* < 0.05)]. AG1 maintained TEER similar to the level of the negative control [−0.1% (*p* = 0.02)]. No differences in inflammatory markers were observed. These in vitro data suggest that acute supplementation with AG1 might stimulate protective effects on GI permeability. These changes may be driven by SCFA production due to the pre-/probiotic properties of AG1, but more research is needed.

## 1. Introduction

The intestinal epithelia, which lines the inner surface of the gastrointestinal (GI) tract, serves several crucial functions in the body. Optimizing the function of the GI epithelia is emerging as a vital concern for human health and well-being [1]. Nutrients such as macronutrients, micronutrients, phytochemicals, and other functional molecules are selectively transported across the epithelial cells and into the bloodstream, providing the body with the necessary components for energy production and overall functioning [2]. Furthermore, this epithelial layer acts as a physical and immunological barrier that prevents the entry of harmful substances such as pathogens, toxins, and undigested food particles from entering the bloodstream and causing systemic inflammatory response [3,4]. Therefore, a well-functioning GI epithelial layer is essential for maintaining overall gastrointestinal health and gut barrier integrity, preventing systemic inflammation and a myriad of negative health conditions [1].

The gut epithelial barrier is comprised of various cell types held tightly together by tight junction (TJ) complexes. The TJ complex includes several proteins, such as claudins, occludins, and zonula occludens (zonulins) proteins. The TJ complex regulates the size and selectivity of the gaps between cells and plays a central role in regulating the paracellular permeability of the barrier [5,6]. As our understanding of GI permeability continues to expand, the body of literature suggests a convincing association between impaired GI permeability and chronic health conditions spanning from autoimmune diseases such as inflammatory bowel disease (IBD) to metabolic disorders, cardiovascular disease, and even neurodegenerative conditions [7,8,9,10]. Although the mechanisms are not completely understood, the aforementioned conditions are likely related to GI permeability through inflammation, a common characteristic of these conditions [7,8,9,10]. Inflammatory cytokines (i.e., TNF-⍺ and IFN-γ) in the gut have negative impacts on TJ proteins, leading to increased GI permeability that may exacerbate systematic inflammation and negatively impact these disease states [9,11]. Beyond diseased populations, exercise-induced GI permeability and cell damage have been linked to impaired nutrient absorption, heightened inflammation, and GI distress [12,13,14,15]. Taken together, there is compelling evidence that mitigating GI damage and permeability is essential for overall health, performance, and the prevention of chronic health conditions [1].

Nutritional interventions to improve and preserve the function and integrity (e.g., of the gut epithelium have garnered attention from practitioners, researchers, and consumers alike [16,17,18,19,20]). AG1^®^ (AG1) is a novel foundational nutrition supplement that contains vitamins, minerals, prebiotics, probiotics, phytonutrients, whole food concentrates, adaptogens, and other functional nutrients. In a previous study utilizing the Simulator of the Human Intestinal Microbial Ecosystem (SHIME^®^) inoculated with feces from healthy donors, we demonstrated the ability of AG1 to undergo fermentation, producing significant increases in acetate, propionate, and total short-chained fatty acids (SCFAs) [21]. SCFAs have been shown to reduce permeability (i.e., improve GI integrity) of the intestinal barrier in part by stimulating the production of TJ proteins, promoting the formation of the protective mucin layer in the gut, and exerting a local anti-inflammatory effect in the gut [22,23,24]. Furthermore, AG1 contains two well-studied probiotic species, *Lactobacillus acidophilus* (3.6 B CFU) and *Bifidobacterium bifidum* (3.6 B CFU), which exhibit the capacity to strengthen the intestinal epithelium through a variety of mechanisms [25,26]. Taken together, it is plausible that due to the combination of ingredients contained in AG1 (e.g., prebiotics, probiotics, phytonutrients) along with initial in vitro data, AG1 supplementation may promote the maintenance of epithelial integrity and function.

Studies examining GI permeability in humans face challenges due to many factors that influence human GI permeability with significant variability among individuals (e.g., genetic factors, diet, physical activity level, lifestyle, and underlying health conditions) [27,28,29]. This variability, along with the inherent limitations (e.g., standardization, invasiveness) of various measurement techniques (e.g., lactulose–mannitol test, endoscopy, plasma biomarkers), can make it challenging to draw conclusions stemming from a nutritional, exercise, or lifestyle intervention [30,31,32,33]. Specifically, these methods are traditionally included in human studies assessing GI permeability and working with human subjects has several challenges (compliance, dropouts, etc.); the lactulose–mannitol test can exhibit significant inter-individual variability due to specimen collection time and disease conditions [34], and endoscopy is invasive and costly. Conversely, Transepithelial Electrical Resistance (TEER) is a widely used model in cell biology and physiology to measure the integrity of epithelial cell layers [35]. TEER is a highly controlled methodology used in vitro that limits the factors that may influence results associated with the other methods discussed above [35]. The TEER measurement assesses the tightness and barrier function of these epithelial cell layers by measuring the electrical resistance across them [35]. An intact and well-functioning epithelial barrier will have high electrical resistance, while a compromised barrier will have low resistance [35]. Utilizing co-cultures of enterocyte-like cells (Caco-2) and THP1 macrophages, an in vitro model for gut epithelial inflammation has been shown to reliably assess the influence of an intervention of GI permeability while elucidating its effect on the local inflammatory response [36,37,38].

The purpose of this investigation was to examine the effects of AG1 on an in vitro model of GI permeability and inflammation. To accomplish this, we co-cultured Caco-2/THP1 cells with fermented AG1 and non-AG1 colonic suspensions from the Simulator of Human Intestinal Microbial Ecosystem (SHIME^®^) model. This design allows for an evaluation of the effect induced by the product and the fermentation-derived metabolites produced by the gut microbiota during the digestive steps on the gut epithelial cells. The primary endpoints of this study were related to gut barrier integrity (TEER) and immune markers (pro- and anti-inflammatory cytokines and chemokines) in an in vitro Caco-2/THP1 co-culture model. We hypothesized that co-culturing Caco-2/THP1 cells with fermented AG1 colonic suspensions following an inflammatory challenge would beneficially impact GI permeability compared to control.

## 2. Materials and Methods

### 2.1. Test Product

AG1 was compared to the blank vehicle for the experiment. Briefly, the blank vehicle in the upper gastrointestinal tract (UGIT) simulation was devoid of AG1 but contained gastric and small intestine media, while the colon simulation contained the UGIT suspension, colonic medium, and fecal inoculum [21]. AG1 (Carson City, NV, USA) is a novel foundational nutrition supplement that contains vitamins, minerals, phytonutrients, probiotics, and prebiotics in powder form. Specifically, AG1 contains 7.2 billion colony-forming units (CFU) of a probiotic blend of *Lactobacillus acidophilus* (UALa-01) and *Bifidobacterium bifidum* (UABb-10). A dose of 12 g per serving is recommended, but a dose of 6 g per reactor was used for this experiment to mitigate physical complications that would potentially affect the biological and mechanical factors of the GI model. Due to biological factors, a supportive media (PD001 [a carbohydrate-depleted background nutritional medium representative for the colon environment; ProDigest, Ghent, Belgium]) was used as a vehicle and was described elsewhere [21]. The list of ingredients present in AG1 is available online [39]. AG1 has undergone third-party verification and evaluation via NSF testing (Ann Arbor, MI, USA) to confirm that the supplement meets strict safety, quality, purity, and label accuracy standards [40].

### 2.2. SHIME^®^ Model, Gastric Phase, Intestinal Phase, and Colonic Simulation

Briefly, we employed the SHIME^®^ model which is jointly registered by ProDigest and Ghent University in Belgium [41]. This model was chosen as it emulates the chemical and physiological conditions of the human gastrointestinal tract to simulate realistic conditions anticipated in humans. AG1 was exposed to a gastric phase in which the test product was subjected to normal stomach physiological conditions. Following the gastric phase, physiological conditions were shifted towards conditions of the duodenum briefly and then transferred to a dialysis membrane to emulate absorption of the digested fraction. The non-absorbed fraction was subsequently transferred to a mixture of colonic medium and human fecal inocula from three healthy adults (normal weight BMI, free from diseases associated with impaired gut microbiome status, and no antibiotic medication used in the prior four months). Colonic simulations were performed under physiological conditions of the proximal colon for 48 h. For more detailed information on the methodology, please refer to our previous publication [21]. The study was conducted in accordance with the Declaration of Helsinki and approved by the Ethics Committee of the University Hospital Ghent (reference number ONZ-2022-0267). It is the metabolic output from the colonic simulation that was applied to the subsequent cell culture experiments.

### 2.3. Caco-2 and THP1-Blue™ Cells

Caco-2 cells (HTB-37; American Type Culture Collection; passage number 48) were maintained in Dulbecco’s Modified Eagle Medium (DMEM) containing glucose and glutamine and supplemented with HEPES and 20% (*v*/*v*) heat-inactivated (HI) fetal bovine serum (FBS).

THP1-Blue™ (InvivoGen, San Diego, CA, USA) cells were maintained in Roswell Park Memorial Institute (RPMI) 1640 medium containing glucose and glutamine, supplemented with HEPES, sodium pyruvate and 10% (*v*/*v*) HI-FBS. Cells were incubated at 37 °C in a humidified atmosphere of air/CO2 (95:5, *v*/*v*).

### 2.4. Caco-2/THP1-Blue Co-Culture Model

The co-culture experiment was performed as previously described [36]. Briefly, Caco-2 cells were seeded in 24-well semi-permeable inserts (0.04 µm pore size) and cultured for 14 days, with three medium changes/week as described [42]. THP1-Blue™ cells were seeded in 24-well plates and treated with PMA (P1585, Sigma-Aldrich, St. Louis, MO, USA) for 48 h [42].

**Figure 1:**
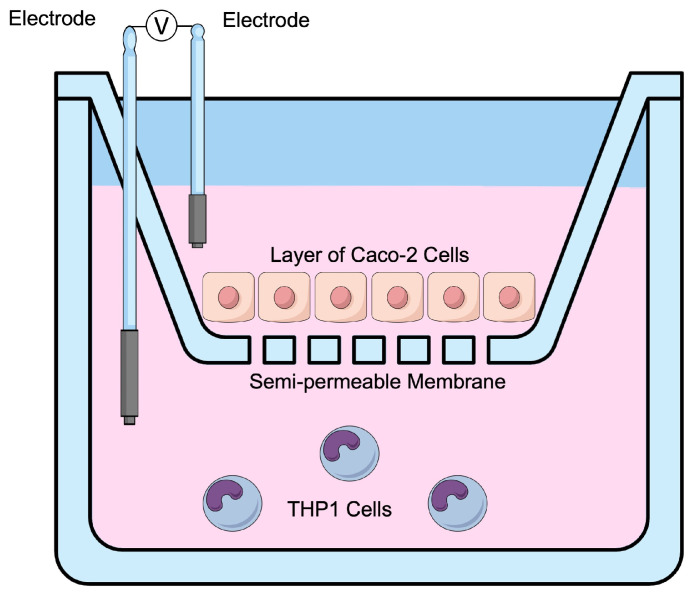
Schematic overview of the Caco-2/THP1-Blue™ co-culture model. Caco-2-bearing inserts were placed on top of the PMA-differentiated THP1-Blue™ cells. Transepithelial electrical resistance (TEER) was measured using electrodes placed in the apical (Caco-2 cells) compartment and basolateral (THP1-Blue cells) compartment. Individually, the sodium butyrate (positive control), Caco-2 complete medium (negative control), AG1 treated fecal inoculum, and blank treated fecal inoculum were added to the apical compartment for 24 h, followed by a 6 h addition of lipopolysaccharide to the basolateral compartment.

Before setting up the co-culture, the TEER of the Caco-2 monolayers was measured (=0 h time point). Then, the Caco-2-bearing inserts were placed on top of the PMA-differentiated THP1-Blue™ cells (Figure 1), as previously described [36,38]. The apical compartment (containing Caco-2 cells) was treated with 12 mM sodium butyrate (NaB) (B5887, Sigma-Aldrich, St. Louis, MO, USA) as a positive control or Caco-2 complete medium (CM) as a negative control. The experimental portion of this model utilized the AG1 and blank-treated fecal inoculum from colonic batch simulation (described above). Briefly, colonic suspensions were collected following the 48 h of colonic simulation, filter-sterilized (0.22 μm), diluted (1:5, *v*/*v*) in CM, and given apically to the co-cultures. Cells were incubated for 24 h, after which the TEER was measured (=24 h time point). Then, the basolateral supernatant was discarded, and cells were stimulated at the basolateral side with CM containing 500 ng/mL ultrapure LPS (tlrl-eklps, *Escherichia coli* K12, InvivoGen, San Diego, CA, USA). After 6 h of LPS stimulation (=30 h of apical treatment of the co-cultures with colonic suspensions), the basolateral supernatant was collected to measure the secretion of anti- and pro-inflammatory cytokines and chemokines (human IL-1β, IL-6, IL-8, IL-10, TNF-α, CXCL10 and MCP-1 by Luminex^®^ multiplex (Thermo Fisher Scientific, Waltham, MA, USA)) and NF-κB activity using the QUANTI Blue reagent (rep-qbs, InvivoGen, San Diego, CA, USA), according to the manufacturer’s instructions. All treatments were performed in triplicate.

### 2.5. Statistics

Samples from the colonic batch incubations were conducted in triplicate as biological replicates for all cell assays. Differences in TEER and immune markers between the blank control and AG1 on the average of all donors were assessed using a two-tailed, paired *t*-test using the average of the individual donors as replicates (*n* = 3). To better visualize the TEER change scores and compare the AG1 and blank-treated colonic suspensions, we normalized the 24 h TEER values to the blank control media (CM) values. The CM value was normalized to 100%, and the same constant was added to all three individual donors 24 h TEER values before calculating the change scores. A *p*-value of < 0.05 was considered statistically significant. All statistics were performed using GraphPad Prism version 10.0.2 for Mac (GraphPad Software, San Diego, CA, USA).

## 3. Results

**Figure 2:**
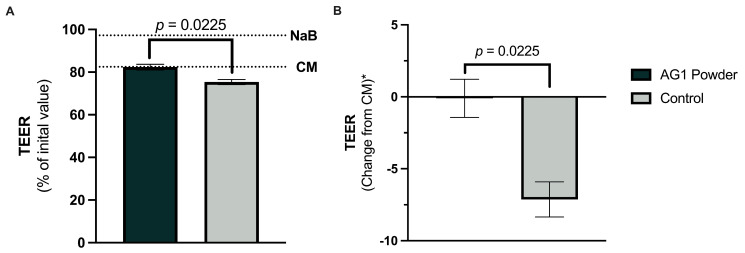
Effects of colonic suspensions on transepithelial electrical resistance (TEER) of the Caco-2/THP1-Blue co-cultures. TEER was measured 24 h after pre-treatment of the co-cultures, and each 24 h value was normalized to the 0 h initial value. Twenty-four-hour TEER values are presented in (**A**), and normalized change scores to complete medium (CM) are presented in (**B**). The dotted line labeled NaB (sodium butyrate) represents the % of the initial value for NaB following 24 h of incubation devoid of fecal inoculum. The dotted line labeled CM represents the experimental control TEER value following the co-culture with activated macrophages devoid of fecal inoculum. Data are plotted as mean ± standard deviations. Statistical analysis included a two-tailed, paired *t*-test using the average of the individual donors as replicates (*n* = 3). * These values are normalized to the CM values.

Following the 24 h culture, a significant endpoint difference in TEER was observed for the AG1 (raw TEER mean and standard deviation: 82.37 ± 1.32) treated colonic suspensions compared to the blank control (raw TEER mean and standard deviation: 75.35 ± 1.22) colonic suspension [mean difference AG1 vs. blank control: 7.02% (95% CI: 2.41, 11.63)] (Figure 2A). The control colonic suspensions decreased TEER [−7.13% (95% CI: −10.17, −4.10)] compared to the CM (activated macrophages challenge in the complete medium devoid of fecal inoculum) control (Figure 2B). Furthermore, AG1 maintained TEER [−0.11% (95% CI: −3.94, 3.18)] at the level of the CM control (Figure 2B).

**Figure 3:**
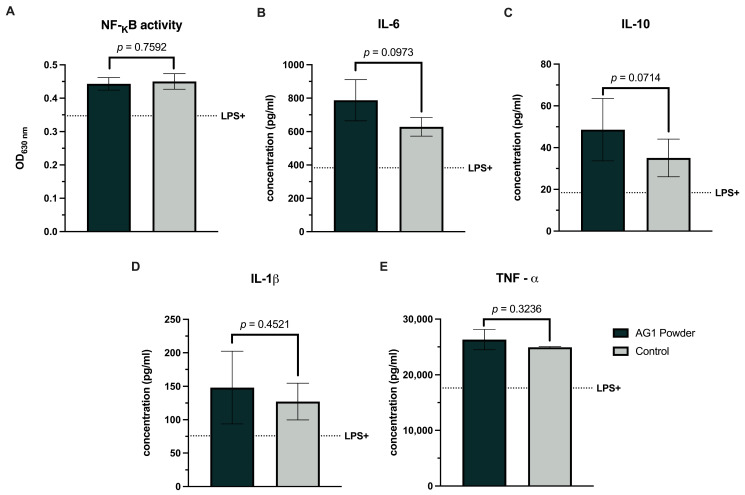
Effects of colonic suspensions on NF-_K_B (**A**), interleukin-6 (**B**), interleukin-10 (**C**), interleukin-1 beta (**D**), and tumor necrosis factor-α (**E**). Immune biomarkers were measured 6 h after LPS treatment on the basolateral side of the Caco-2/THP1-Blue co-cultures after pre-treatment of the apical side for 24 h with the colonic suspensions. The black dotted line labeled LPS+ represents the experimental control value following the LPS challenge devoid of colonic suspension. Data are plotted as mean ± standard deviations. Statistical analysis included a two-tailed, paired *t*-test using the average of the individual donors as replicates (*n* = 3).

**Figure 4:**
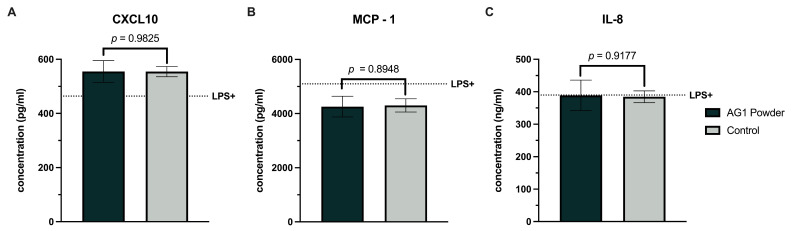
Effects of colonic suspensions on CXCL10 (**A**), monocyte chemoattractant protein-1 (**B**), and interleukin-8 (**C**). Immune biomarkers were measured 6 h after LPS treatment on the basolateral side of the Caco-2/THP1-Blue co-cultures after pre-treatment of the apical side for 24 h with the colonic suspensions. The black dotted line labeled LPS+ represents the experimental control value following the LPS challenge devoid of colonic suspension. Data are plotted as mean ± standard deviations. Statistical analysis included a two-tailed, paired *t*-test using the average of the individual donors as replicates (*n* = 3).

AG1-treated and control colonic suspensions increased NF-κB activity compared to the LPS+ control (Figure 3A). However, no significant differences were observed between the AG1 and control. Similarly, both colonic suspensions increased the anti-inflammatory (Figure 3B,C) and pro-inflammatory cytokines (Figure 3D,E) relative to the LPS+ control. A trend was observed with higher values for IL-6 (*p* = 0.0973) and IL-10 (*p* = 0.0714) for AG1 vs. blank control fecal inoculum. Results for the chemokines were variable, with CXCL10 non-significantly increasing (Figure 4A) and no change in MCP-1 or IL-8 (Figure 4B,C, respectively) for AG1 vs. control.

## 4. Discussion

We aimed to assess the effects of AG1 and the metabolites produced following 48-h of colonic fermentation on gut wall functioning and immune markers following an inflammatory challenge using a Caco-2/THP1 co-culture model. Our results suggest that colonic fermentation of AG1 protected against inflammation-induced barrier disruption compared to blank control colonic suspensions. We did not observe between-group differences in any of the measured pro- or anti-inflammatory biomarkers following the LPS challenge. The protective aspects of gut barrier function in the AG1 group compared to the blank control, following activated macrophages-induced inflammatory challenge, were likely driven by the fermentation of prebiotics [43], subsequent increases in SCFAs [44], and changes to the gut microbial community from pre- and probiotics [45,46].

The main finding of this investigation demonstrated that AG1 improved gut barrier function by attenuating intestinal permeability following an inflammatory challenge. This can likely be explained by our previous work [21], which reported significant increases in SCFA production, a major byproduct of microbial fermentation [47], following AG1 treatment. Specifically, total SCFAs, propionate, and acetate were significantly increased during the 0–24 h and 0–48 h timepoint for AG1 compared to the blank control. A donor-specific effect for butyrate was observed where butyrate was higher at all three time points (0–1 h, 0–24 h, and 0–48 h), yet only statistically significant in 2 of the 3 donors [48]. The current experiment used stool from the same healthy donors in our previous studies [21,48], and thus, it is reasonable to expect similar metabolic conditions in the current experiment. SCFAs have been shown to modulate host health via tissue-specific pathways like glucose homeostasis, immunomodulation, and obesity, but the most compelling impact is on gut barrier integrity [49]. Data from animal (mice, rats, and pigs) studies using challenge models of disease (chronic kidney disease, autoimmune hepatitis, diet-induced metabolic dysfunction, peritonitis, diarrhea, and acute liver failure) demonstrate that SCFAs restore normal barrier function [50,51,52,53] and maintain epithelial integrity [54,55] through their beneficial effects on tight junction proteins. Butyrate, one of the SCFAs, is an important energy source for intestinal epithelial cells [56] and is known to regulate TJ assembly [57], thus positively enhancing the intestinal barrier. Further, the immunomodulatory effect of the SCFAs has also been known to enhance the intestinal barrier by regulating inflammatory processes and preserving the functionality of the TJ proteins [58]. Taken together, it is reasonable to conclude that the beneficial impacts AG1 had on the intestinal barrier in this study are potentially, in part, mediated by the SCFAs produced during the fermentation of AG1.

In addition to prebiotics, probiotics have been shown to exert a significant impact on gut barrier function [59]. Several species, like *Bifidobacterium bifidum* [25] and *Lactobacillus acidophilus* [26,60], have been reported to improve TEER, positively influence TJ proteins, and restore epithelial function in both animal and human cell models. Additionally, these probiotics are important in the production of SCFAs [61,62] which can have indirect beneficial effects on the gut barrier function through previously described mechanisms. There are, however, some nuances with probiotics that can influence their efficacy. Many studies report that the number of viable bacteria that survive the GI tract and make it to the colon diminishes due to a plethora of factors beyond the scope of this manuscript [63]. Generally speaking, however, *L. acidophilus* and *B. bifidum* are reported to survive the journey as they are metabolically equipped to handle harsh environments [64,65]. Of course, the survivability and subsequent health benefits likely to arise from the probiotic species are also dependent on the dose the frequency of dosing. Based on the label claim and corrected for the dosing used in the model, a total of 3.6B CFU underwent digestion and subsequent delivery to the colonic microbiota along with other undigested components of the product (likely protein and fibers which were not quantified) only one time (acutely). Other aspects of the probiotic, like the type of bacteria and even the specific strain can also impact survivability as well as efficacy of the probiotic.

AG1 is a synbiotic blend containing prebiotic phytonutrients and a probiotic blend of *Lactobacillus acidophilus* (UALa-01) and *Bifidobacterium bifidum* (UABb-10). Direct effects on gut barrier integrity from cell models have been evaluated by Hsieh et al., 2015 [25] and Al-Sadi et al., 2021 [26] where they demonstrated significant improvements in TEER following *B. bifidum* or *L. acidophilus* supplementation, respectively. A study assessing the effects of *L. acidophilus* DDS-1 in young and aging C57BL/6J mice demonstrated increases in acetate, propionate, and butyrate [62]. In 103 adults with chronic constipation, supplementation with 2 billion CFUs *B. bifidum* (CCFM16) for 28 days demonstrated increased SCFA concentrations compared to control, specifically acetate and butyrate [61]. Therefore, while the maintenance of GI epithelial function in the current study may be largely attributed to the prebiotic influence and production of SCFAs, it is likely that the probiotic species contained in AG1 in part contributed to the heightened TEER measurements. Future in vitro studies with larger sample sizes are needed to confirm these findings, and in vivo studies are necessary to determine how AG1 impacts SCFA production in humans. Moreover, studies assessing the additional mechanism of action (e.g., tight junction proteins) are needed to understand how AG1 exerts beneficial effects on the intestinal barrier.

This study had several limitations that must be taken into consideration when interpreting the results. This was a proof-of-concept study with a small sample size (*n* = 3) and an acute intervention phase. The increase in anti-inflammatory cytokines (trend for significance) following AG1 treatment is of interest to further understand the effect of AG1 on the intestinal barrier, but the small sample sizes led to large variability in the pro- and anti-inflammatory endpoints and larger studies are needed to adequately assess the immunomodulatory effects of AG1. Similarly, this was an acute investigation assessing an acute dose of AG1 on the intestinal barrier and immunomodulation. Longer-term studies are needed to confirm these findings and examine how chronic ingestion of the synbiotic may affect gut barrier integrity and the immune response to both acute (e.g., exercise, medication) and chronic stressors (e.g., stress, poor dietary choices, disease). That being said, the TEER model employed in this study is well-controlled and a reliable assessment of GI integrity in vitro. Another strength of this study design is that the colonic suspensions were taken from the SHIME model, where the test products underwent a complete simulation of the human gastrointestinal tract before being added to the fecal inoculum. This allowed for physiologically relevant concentrations of the test product’s metabolites to be used for incubation and subsequent exposure to epithelial cells in the TEER experiment.

The data from this experiment, when paired with previous work, suggest that acute supplementation with AG1 might exert protective effects on the intestinal barrier integrity, likely via SCFA production. However, these findings must be further investigated using larger sample sizes in vitro and clinical settings.

## Data Availability

Upon a reasonable request, the corresponding author will provide accessible data; however, privacy concerns may restrict the release of certain data.
